# Supplementary material for: CD74 and CD44 Expression on CTCs in Cancer Patients with Brain Metastasis
Source: Int J Mol Sci. 2021 Jun 29;22(13):6993. doi: 10.3390/ijms22136993 (PMC8268634; doi:10.3390/ijms22136993)
Supplement: Supplementary file 1 [file ijms-22-06993-s001.zip › ijms-1262127-supplementary.pdf]

Supplementary material

Three supplementary figures and the according figure legends describing the gating strategy to identify CTCs, CD74 and CD44 expression on CTC enriched with NSCLC and melanoma patients' blood and additional CNV profiles showing the heterogeneity of CTCs of P6.

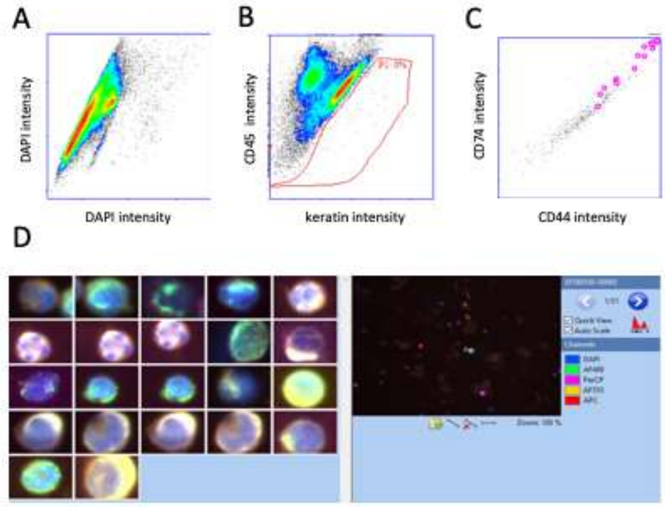

Figure S1: Gating strategy for identifying CTCs by Xcyto®. (A) Cells were recognized by positive DAPI staining and (B) CTCs were identified as keratin-positive and CD45-negative. (C) Displaying of CD74 and CD74 intensity shows linear correlation of intensities. (D) CTC gallery obtained from the red gate in (B).

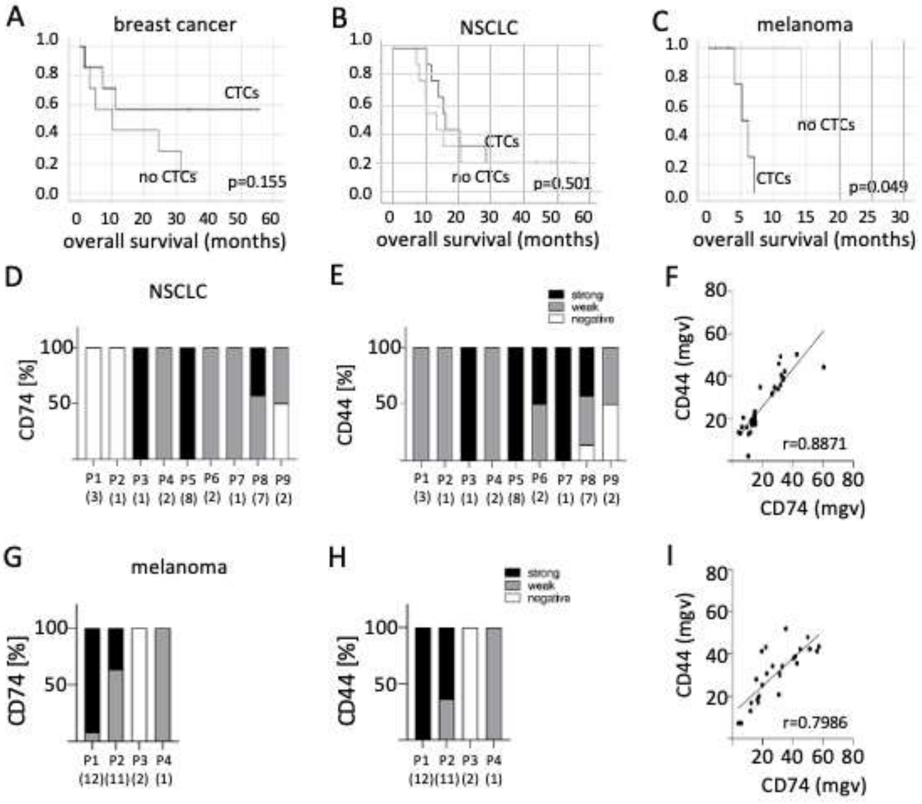

Figure S2: CD74 and CD44 expression on CTCs from breast cancer, NSCLC and melanoma patients with BM. (A-C) CTC count had no effect on the overall survival for breast cancer (p=0.155) and NSCLC patients (p=0.501), whereas in melanoma, a positive CTC status predicts a worse overall survival (p=0.049). (D-F) CTCs from NSCLC patients showed a heterogeneous and mainly positive protein expression for CD74 and CD44. Correlation analysis revealed a positive association of both proteins on NSCLC CTCs. (Pearson's r=0.8871). (G-I) In melanoma, the expression pattern of CD74 and CD44 is also heterogeneous and comparable in enriched CTCs with a slightly weaker correlation of both proteins compared with NSCLC-BM CTCs (Pearson's r=0.7986). (D, E, G, H: Number in parenthesis represents the number of CTCs per patient) (mgv = mean grey value.)

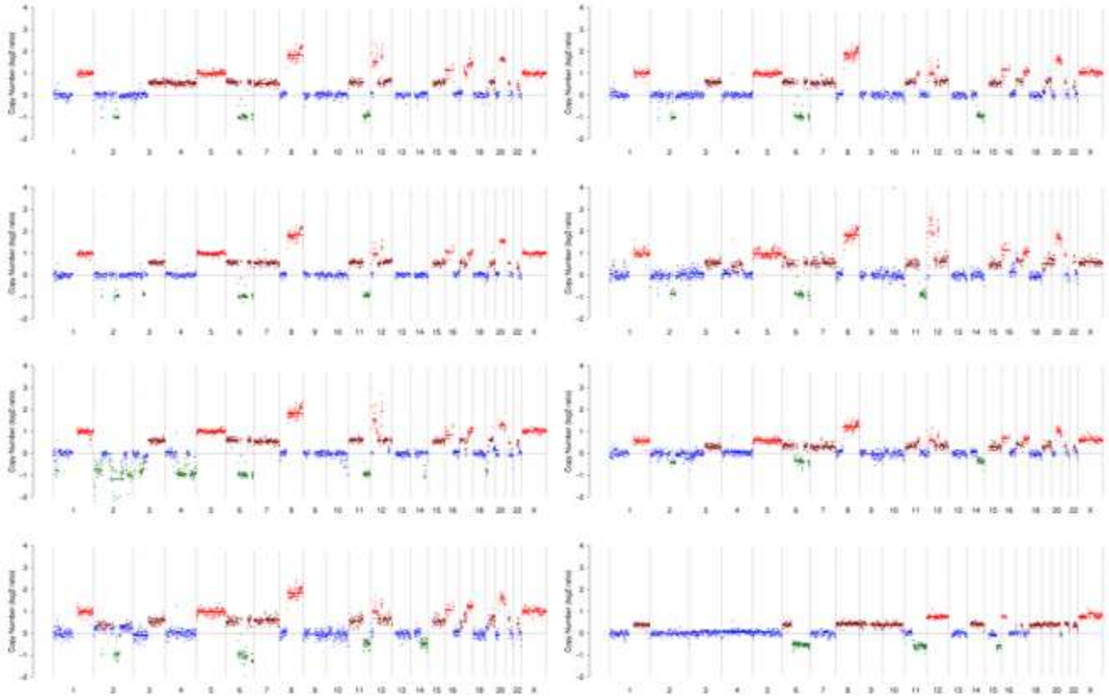

Figure S3: CNV plots of 8 single CTCs of breast cancer P6.
